# An ethnographic study of group decision making to understand and improve how trial steering committees contribute to trial conduct

**DOI:** 10.1186/1745-6215-14-S1-O79

**Published:** 2013-11-29

**Authors:** Anne Daykin, Ali Heawood, Athene Lane, Rhiannon Macefield, Carrol Gamble, Sharon McCann, Gillian Shorter, Matthew R Sydes

**Affiliations:** 1 School of Social and Community Medicine, University of Bristol, Bristol, UK; 2 Department of Biostatistics, University of Liverpool, Liverpool, UK; 3 University of Aberdeen, Aberdeen, UK; 4 University of Ulster, Ulster, UK; 5 MRC Clinical Trials Unit, London, UK; 6 MRC Clinical Trials Unit Hub for Trials Methodology Research, London, UK

## Introduction

According to the MRC Guidelines for Good Clinical Practice, the role of Trial Steering Committees (TSCs) is to provide overall supervision of a trial. While the HTA DAMOCLES project resulted in a charter for Data Monitoring Committees, there is currently little empirical evidence regarding how TSCs oversee trials and make decisions about trial conduct.

## Methods

Using ethnographic methods, we are exploring how TSCs make decisions regarding trial conduct in a purposive sample of trials across registered trials units in the UK, selected to represent a range of trial designs, interventions and oversight structures. Non-participatory observation of TSCs will be triangulated by follow-up interviews with committee members and evaluations of trial documentation. Data collection is ongoing.

## Results

Preliminary results suggest that the TSC Chair is central to decision making and the effectiveness of the committee. However, the Chair’s scope and focus is partly framed by reports and information provided by the trial teams. TSC members agree to take on the role for various reasons including reciprocal altruism. Initial evidence also implies that the remit and structure of TSCs varies according to the trial funder. More detailed results will be presented at conference.

## Conclusion

It is emerging that decision making within TSCs can potentially be influenced by the Chief Investigator but can be countered by the skill of the Chair in gaining consensus decisions and the will of the members to do the right thing. Recommendations from this research and a parallel quantitative study will contribute to updating the MRC Guidelines.

